# UPLC–ESI–QToF–MS Profile of Hydroalcoholic Extract of *Persea cordata* (Meisn.) and its Antioedematogenic and Antinociceptive Effects in Mice

**DOI:** 10.1002/cbdv.202502441

**Published:** 2025-10-24

**Authors:** Valfredo Schlemper, Daniela Hemsing, Julia Elisabett K. Bolsonello, Susana R. de Mello Schlemper, Tiago Tizziani, Lutuima A. Capangue Neto, Louis P. Sandjo

**Affiliations:** ^1^ Veterinary Medicine Undergraduate Degree Federal University of Fronteira Sul (UFFS) Realeza Brazil; ^2^ Graduate Degree Program in Health, Welfare, and Sustainable Animal Production of Fronteira Sul Federal University of Fronteira Sul (UFFS) Realeza Brazil; ^3^ Department of Chemistry Federal University of Santa Catarina (UFSC) Florianópolis Brazil

**Keywords:** anti‐oedematogenic activity, pain, paw oedema, UPLCMS profile, *Persea cordata*

## Abstract

This study aimed to establish the chemical profile and evaluate the anti‐oedematogenic and analgesic activities of the hydroalcoholic extract (HEPC) of *Persea cordata* bark. In the oedema model induced by carrageenan (CAR), HEPC, exhibited inhibitory effects after 60 min, with maximum inhibition (MI) reaching 89.55 ± 3.17% at 240 min (dose of 100 mg/kg). At doses of 30 and 100 mg/kg, it showed similar effect as meloxicam (3 mg/kg) after 24 h of treatment. In the model of capsaicin‐induced oedema (PCS), HEPC displayed MI of 86.23 ± 8.6% during 24 h at 100 mg/kg while meloxicam displayed similar activity at 3 mg/kg. HEPC UPLC–ESI–MS/MS revealed the presence of 10 compounds with the predominant belonging to lignans. Our results suggest that orally administered HEPC has a non‐specific anti‐oedematogenic and antinociceptive effects, however, it may be related to the presence of anti‐inflammatory metabolites that can regulate redox activity and inflammatory mediators.

## Graphical abstract

Hydroalcoholic crude extract of *Persea cordata* showed in its composition, the presence of lignans based on LCESIMS data. This crude extract displayed anti‐oedematogenic and antinociceptive activities *in vivo*.

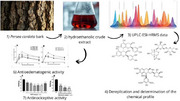

## 1 Introduction

Inflammatory pain is a sensitive perception caused by noxious stimuli, and medical control has always been a challenge. In general, the available pain relief drugs show limitations because of adverse events such as sedation, gastrointestinal and kidney disorders and addiction, among other side effects [1]. Some of these drugs have restrictions concerning their administration, and others face a reduced half‐life, which hinders the treatment of pain and inflammation [2].

Therefore, the search for new anti‐inflammatory and analgesic agents remains important. Herbal medicine has significantly contributed as an alternative medicine to cure numerous illnesses and restore human wellbeing [3]. Medicinal plants are still a great source of new leads from which numerous small molecules have proved to possess analgesic and anti‐inflammatory properties [4, 5].

The species *Persea cordata* (Meisn.) (Lauraceae) also botanically identified as *Persea major*, *Persea pyrifolia* Spreng, *P. pyrifolia* Nees and *Persea willdenovii* Kosterm is a native tree of the Araucaria Forest, popularly known as ‘*pau‐andrade*’, ‘*abacateiro‐do‐mato*’, ‘*maçaranduba*’ and ‘*canela‐rosada*’ [6, 7, 8]. Its bark is used topically in popular medicine, mainly in the rural areas, for the treatment of skin diseases and wounds [7]. It is also (two to three pieces of the bark) prepared as an infusion in 1 L of water and consumed as a tea to treat ulcer and gastric illnesses [8, 9].

Other previous studies demonstrated that the plant possesses antibacterial [10, 11], anti‐oedematogenic [12], antispasmodic [13, 14] and gastroprotective properties [14].

As far as we can see in the literature, its analgesic effect has never been investigated, although ethyl acetate and n‐butanol fractions of the plant exhibited anti‐oedematogenic action in the microvascular leakage in rat skin by intravenous route [12]. Since pain is one of the cardinal signs of inflammation, with a direct link to inflammatory/algogenic mediators, it is not uncommon for some drugs to exhibit both anti‐inflammatory and analgesic effects.

As medication efficacy not only relies on its pharmacological property but also on the bioavailability and bio‐absorption, which depend on the administration route [15], this work was designed to treat mice by oral administration of the hydroalcoholic crude extract. This administration route was based on the traditional use of the studied plant.

Therefore, we herein report through pre‐clinical studies, the anti‐oedematogenic and antinociceptive effects of bark hydroalcoholic extract of *P. cordata* in mice by oral route. We also report the chemical profile of the bark crude extract established by using data from liquid chromatography coupled to mass spectrometry analyses.

## 2 Results and Discussion

Plants of the Lauraceae family are found to be rich in substances with large phytochemical interest. Based on ethnopharmacological and ethnoveterinary reports, *P. cordata* has a long history in popular medicine as an anti‐inflammatory agent in skin diseases, mainly in equine [14]. Before evaluating the biological activity of this plant species, it seemed important to establish its chemical profile by UPLC–ESI–MS to acknowledge the constituents responsible.

### 2.1 UPLC–ESI–HRMS Data

**FIGURE 1:**
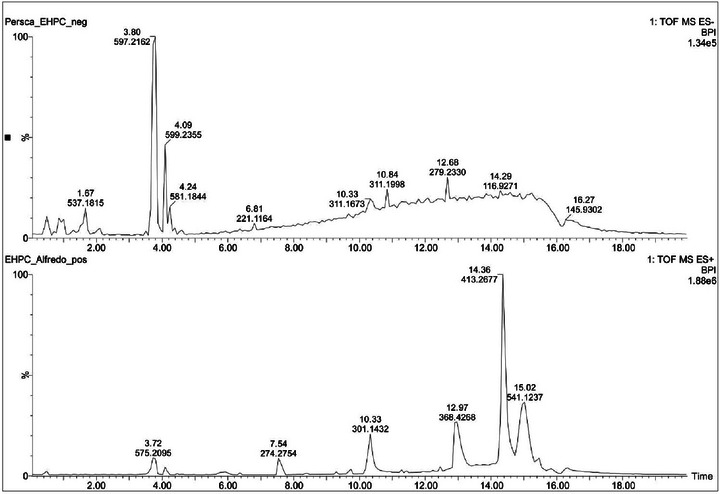
UPLC–ESI–HRMS data obtained from positive and negative ionization modes, of the hydroalcoholic extract of *Persea cordata*.

UPLC–ESI–MS data of HEPC showed in its phytochemical composition 10 secondary metabolites in positive and negative ionization modes (Figure 1) as described in Table 1. This is the first research done with the bark of *P. cordata*, which allowed the identification of some unpublished compounds.

**TABLE 1:** Proposal of the chemical constituents obtained from UPLC–ESI–QToF–MS data of *Persea cordata*.

*t*_R_ (min)^a^	Mass value (*m*/*z*) and molecular formula (error)		Fragment ions (*m*/*z*)		Proposed structure
	ESI^−^	ESI^+^	ESI^−^	ESI^+^	
0.46	885.2394 [C_42_H_46_O_21_−H]^−^ (−6.70)	887.2409 [C_42_H_46_O_21_+H]^+^, 909.2432 [C_42_H_46_O_21_+Na]^+^ (0.30)	577.1318, 289.0712, 269.0440	289.0698, 291.0870 579.1495, 741.2051	Procyanidin B2 rutinoside or glucopyranosyl‐rhamnopyranosyl procyanidin B2
0.86	885.2394 [C_42_H_46_O_21_−H]^−^ (−6.70)	—	577.1318, 289.0712, 269.0440	—	Isomer of procyanidin B2 rutinoside or glucopyranosyl‐rhamnopyranosyl procyanidin B2
1.67	537.1815 [C_22_H_34_O_15_−H]^−^ (−0.83)	—	—	—	NI
3.50	563.1956 [C_23_H_34_O_13_−HCO_2_]^−^ (−3.54)	—	—	—	NI
3.80	597.2162 [C_27_H_36_O_12_+HCO_2_]^−^ (−3.57)	575.2094 [C_27_H_36_O_12_+Na]^+^ (1.82)	419.1683, 404.1472, 373.1284, 359.1515, 233.0815, 202.0614	—	Lyoniside or nudiposide
4.09	599.2355 [C_27_H_38_O_12_+HCO_2_]^−^ (2.54)	577.2269 [C_27_H_38_O_12_+Na]^+^ (1.39)	421.1839, 406.1636, 388.1479	—	Ssioriside
4.24	581.1844 [C_27_H_34_O_14_−H]^−^ (−4.53)	—	273.0746, 167.0348	—	Naringin dihydrochalcone or phloretin 2‐O‐α‐l‐rhamnopyranosyl‐(1→6)‐β‐d‐glucopyranoside
4.49	497.2742 [C_26_H_42_O_9_−H]^−^ (1.73)	—	317.2097	—	Sagittarioside b or suavioside F
4.60	509.1998 [C_24_H_32_O_9_−HCO_2_]^−^ (−4.88)	487.1959 [C_24_H_32_O_9_+Na]^+^ (3.07)	—	—	Related to sakuraresinol
14.36	—	413.2677 [C_24_H_38_O_4_+Na]^+^ (2.23)	—	149.0244	Dialkyl phthalate

^a^ Retention time.

The first metabolite with retention time (*t*_R_) 0.46 min was detected with *m*/*z* 885.2394 [C_42_H_46_O_21_−H]^−^ and 909.2432 [C_42_H_46_O_21_+Na]^+^. The positive mode tandem mass spectrum showed the fragment ions *m*/*z* 741.2051 and 579.1495. These fragments were formed after the precursor lost a deoxyhexose moiety (146 Da) and two sugar moieties (146 and 162 Da), respectively. The peak *m*/*z* 579.1495 was identified as procyanidin B2, as it produced two monomers with *m*/*z* 289.0698 and 291.0870 related to dehydrocatechin and catechin, respectively. The tandem mass spectrum obtained from negative mode ionisation also showed a fragment ion corresponding to procyanidin B2 with *m*/*z* 577.1318. The fragmentation of the aglycon to afford catechin (*m*/*z* 289.0712) and dehydrocatechin (*m*/*z* 269.0440). Based on the abovementioned data, the structure of this metabolite was assigned as a procyanidin B2 rutinoside derivative. Another possibility of the structure assignment would be the sugar moieties rhamnose and glucose separately attached to two procyanidin B2 functionalities. Therefore, the structure of this metabolite can unambiguously be assigned by using NMR data. Another isomer of the metabolite was observed at *t*_R_ 0.86 min with the same mass value *m*/*z* 885.2394. A literature search that revealed natural products related to procyanidin B2 attached to glucose and rhamnose have never been reported. Only two functional isomers were reported in the literature, but the aglycone was related to gambiriin C [16]. Procyanidins were already reported in *Persea* species such as *Persea americana* [17, 18].

The peak at 1.67 min with *m*/*z* 537.1815 in negative mode ionization corresponds to the molecular formula C_22_H_34_O_15_. The tandem mass spectrum of this metabolite did not afford any fragment ions that could consistently lead to a structure assignment. The compound at 3.50 min with *m*/*z* 563.1956 [C_23_H_34_O_13_−HCO_2_]^−^ also did not yield any fragment ions that could allow a structure determination.

The metabolite at *t*_R_ 3.80 min with *m*/*z* 597.2162 [C_27_H_36_O_12_+HCO_2_]^−^ and 575.2094 [C_27_H_36_O_12_+Na]^+^ provided in its tandem mass spectrum in negative mode ionization fragment ions *m*/*z* 419.1683, 404.1472, 373.1284, 359.1515, 233.0815, 202.0614, and 153.0533. The ion *m*/*z* 419.1683 was formed after the precursor [M−H]^−^ lost a pentose (132 Da) and was identified as lyoniresinol [19]. The ion *m*/*z* 419.1683 differs from *m*/*z* 404.1472 by 15 Da, suggesting a radical loss of a CH_3_ group. The fragment *m*/*z* 373.1284 was furnished after *m*/*z* 419.1683 lost H_2_CO (30 Da) and CH_4_ (16 Da). The elimination of two molecules of H_2_CO (60 Da) from *m*/*z* 419.1683 afforded *m*/*z* 359.1515. The ion *m*/*z* 419.1683 also eliminated 2,6‐dimethoxyphenol (154 Da) and CH_3_OH (32 Da) to furnish *m*/*z* 233.0815. The homolytic cleavage of CH_3_O group from *m*/*z* 233.0815 produced *m*/*z* 202.0614. The aforementioned information led to the structure of two aryltetralin lignans, namely, lyoniside and nudiposide. They were previously identified from *Canarium bengalense* (Burseraceace) [20]. Search on *Persea* species revealed that aryltetralins were previously found in *Persea lingue* [21]. Lyoniresinol, the genin and lyoniside were previously obtained from *P. americana* [19].

Another lignan was detected at *t*_R_ 4.09 min with *m*/*z* 599.2355 [C_27_H_38_O_12_+HCO_2_]^−^ and 577.2269 [C_27_H_38_O_12_+Na]^+^. The precursor [M−H]^−^ produced the fragment *m*/*z* 421.1839 corresponding to 5,5′‐dimethoxysecoisolariciresinol (DMSL) previously identified from *Apollonias barbujana* (Lauraceae) [22]. It showed fragmentation patterns similar to those of lyoniresinol with a radical cleavage and a loss of the CH_3_ group to produce *m*/*z* 406.1636. The ion *m*/*z* 388.1479 was formed after DMSL ion lost CH_3_OH (32 Da) and a radical hydrogen (1 Da). The abovementioned data allowed the identification of this metabolite as ssioriside previously obtained from *Lindera umbellata*, a Lauraceae [23].

The peak at 4.24 min showed *m*/*z* 581.1844 [C_27_H_34_O_14_−H]^−^, which produced fragment ions *m*/*z* 273.0746 and 167.0348. The disaccharide cleavage (deoxyhexose and hexose: 308 Da) from the precursor afforded *m*/*z* 273.0746; A loss of *p‐*quinone methide caused by H‐rearrangement and Cα‐Cβ bond cleavage in *m*/*z* 273.0746 afforded *m*/*z* 167.0348. Analysis of these data led to the structures of naringin dihydrochalcone and phloretin 2‐O‐α‐l‐rhamnopyranosyl‐(1→6)‐β‐d‐glucopyranoside. Naringin dihydrochalcone is an artificial sweetener obtained from the chemical transformation of hesperidin and the second metabolite was previously identified from *Malus crabapples* (rosaceae) and *Combretum griffithii* (combretaceae) [24, 25, 26].

The peak at *t*_R_ 4.49 min with *m*/*z* 497.2742 corresponded to the molecular formula [C_26_H_42_O_9_−H]^−^. It gave in its tandem mass spectrum the fragment ion *m*/*z* 317.2097 after losing a hexose (180 Da). This fragment corresponded based on literature search to kaur‐16‐en‐18‐oic acid. The structures of sagittarioside b and suavioside F were assigned to this compound. Kaurane diterpenes were previously reported in *Lauraceae* species alongside isoryanodanes [27].

The literature search of the peak at 4.60 min with *m*/*z* 509.1998 [C_24_H_32_O_9_−HCO_2_]^−^ and *m*/*z* 487.1959 [C_24_H_32_O_9_+Na]^+^ led to the structure of a neolignane identified as sakuraresinol. However, this metabolite has never been reported in a *Lauraceae* species but was identified in *Eurya japonica* (Theaceae) [28]. Compounds with the same 8,3′‐neolignan skeleton were already reported in *Lauraceae* species such as *Licaria aritu*, *Nectandra amazonum* and *Machilius odoratissima* [22].

The structure of dialkyl phthalate was assigned to the peak at 14.36 min with *m*/*z* 413.2677 [C_24_H_38_O_4_+Na]^+^. This precursor showed in its tandem mass spectrum a single fragment *m*/*z* 149.0244 corresponding to the cation phthalate. The alkyl group could not be consistently identified based on the MS data. However, its structure is related to that of a diisooctyl group. No previous report indicated the presence of this compound in *Lauraceae* species.

The presence of diterpenes and polyphenols, including flavonoids and lignans, may suggest why this plant is widely used as a folk medicine for the treatment of wounds and skin diseases. Compounds from these classes are known to possess anti‐inflammatory and analgesic properties [29, 30].

### 2.2 Paw Oedema Studies

#### 2.2.1 Paw Oedema Induced by Carrageenan

**FIGURE 2:**
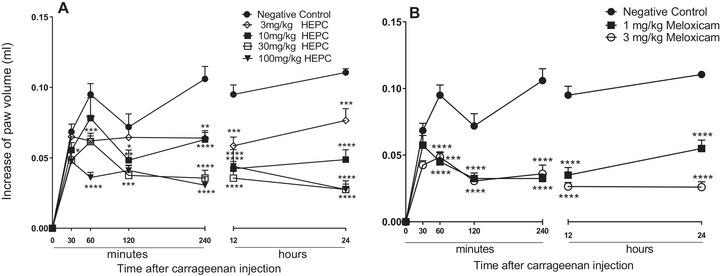
Mean inhibitory effect of the hydroalcoholic extract of *Persea cordata* (HEPC, A) and meloxicam (B) on the oedema induced by carrageenan in the hind limb of the mice. The symbols represent an average of five to six experiments, and the vertical lines represent the SEM. *p < 0.05, **p < 0.01, ***p < 0.001, ****p < 0.0001 in the Dunnett test.

In the “paw oedema” test, induced by carrageenan (CAR) in mice, an intense increase in volume was observed in the limbs of animals when treated with the negative control (NC) throughout the oedema recording process, after 30, 60, 120, 240 min and 12 and 24 h of application of 1% CAR (Figure 2).

The treatment of animals with HEPC promoted a significant and dose‐dependent inhibition of the oedema on the limbs during the entire oedema measuring time. The biggest inhibition was registered at 100 mg/kg, after 240 min of application of a phlogistic agent, with an oedema inhibition value of 89.55 ± 3.17%. The dose of 30 mg/kg of HEPC, significantly exhibited inhibitory effect within 24 h reaching 82.25 ± 3.82% MI, and revealing the long‐term effect of the extract.

It is possible to observe that HEPC significantly inhibited oedema formation starting from M2 (60 min) and that at M1 (30 min) after CAR administration, only the 100 mg/kg dose showed a statistically significant difference (*p* < 0.05) compared to NC (Figure 2A,B). The doses 30 and 100 mg/kg presented pronounced inhibition during the mentioned moments (Figure 2A). The inhibition profile was similar to meloxicam for 24 h after the CAR application (Figure 2B).

CAR releases various neurotransmitters in the biphasic form that sequentially produce inflammatory responses. In the first hour, the CAR releases neurotransmitters such as histamine, serotonin, bradykinin, and neuropeptide [31]. The second stage, which occurs 1 h after administration, involves the release of mediators such as bradykinin and prostaglandins, which play a role in the development of CAR‐induced paw oedema [32]. The inflammation reaches its peak approximately 3 h after treatment (second stage), which is sustained by the overproduction of prostaglandins, followed by the release of cytokines and cellular infiltration [33]. It was with CAR that HEPC exhibited the most significant inhibition of oedema. The results of our studies show that the HEPC has an inhibitory effect in both the early and late stages of the test. The inhibitory profile was similar to meloxicam up to 24 h after the CAR injection (Figure 2B). This suggests that HEPC has significant and long‐lasting anti‐inflammatory activity, involving the inhibition of one or more intracellular signalling pathways involved in the CAR‐induced inflammatory response.

#### 2.2.2 Paw Oedema Induced by Capsaicin

**FIGURE 3:**
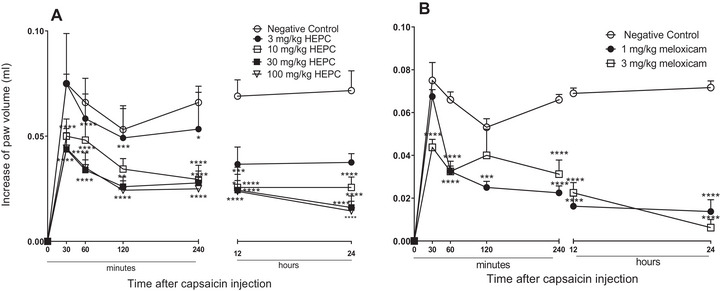
Mean inhibitory effect hydroalcoholic extract of *Persea cordata* (HEPC, A) and meloxicam (B) on capsaicin‐induced oedema in the hind limb of the mice. Each group (dose) represents an average of five to six animals, and the vertical lines indicate the SEM. It significantly differs from the negative control (NC) *p < 0.05, **p < 0.01, ***p < 0.001, ****p < 0.0001, no teste de Dunnett.

In the paw oedema induced by PCS, animals treated with HEPC (10–100 mg/kg) had their oedema reduced significantly and in a dependent dose manner, when compared to NC (Figure 3A). HEPC was able to inhibit PCS‐induced oedema within the first 30 min at doses of 3, 10, 30, and 100 mg/kg, and this effect persisted throughout the entire observation period (Figure 3A).

Comparing the inhibition percentages of HEPC and PC, a similarity in effect can be observed. HEPC showed an MI of 86.23 ± 8.6% during 24 h at a dose of 100 mg/kg, while meloxicam showed an MI of 91.39 ± 6.3% at the 24‐h mark at a dose of 3 mg/kg. In Figure 3B, it can be observed that meloxicam, administered 1 h before the application of the phlogistic agent, significantly always inhibited oedema formation, with both doses being effective in inhibiting PCS‐induced oedema.

PCS, a pungent alkaloid obtained from peppers of the genus *Capsicum* sp. releases neuropeptides from collateral afferent sensory terminals, contributing to neurogenic inflammation. The P substances, neurokinin A, neurokinin B, and the peptide related to calcitonin gene (CGRP), are fundamental neuropeptides in the induction of extravasation of proteins and vasodilation [34]. HEPC significantly inhibited the oedema induced by the PCS or by the neuropeptides released by it, suggesting that HEPC suppresses neurogenic inflammation and, consequently, neurogenic pain.

#### 2.2.3 Paw Oedema Induced by Dextran

**FIGURE 4:**
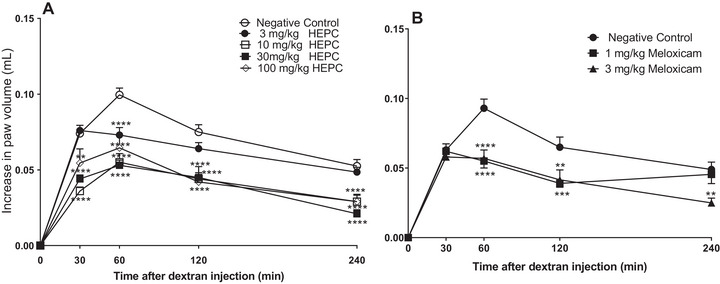
Mean inhibitory effect of hydroalcoholic extract of *Persea cordata* (HEPC, A) or meloxicam (B) on dextran‐induced oedema in the hind limb of the mice. Each group (dose) represents the mean of five to six animals, and the vertical lines indicate the SEM (standard error of the mean). Significantly different from the negative control (NC), **p < 0.01, ***p < 0.01, ****p < 0.0001, in Dunnett's test.

Paw oedema induced by DEX presented oedema different from CAR and PCS, and did not present a second stage (3–4 h post‐treatment), and is usually associated with the production of prostaglandins and leukocyte infiltration [35]. HEPC displayed inhibitory effect at all analysed moment of the DEX‐induced oedema, and the dose with the highest inhibition level was 30 mg/kg with MI of 56.32 ± 4.77 (30 min) and 77.88 ± 4.72% (60 min), respectively (Figure 4A). It suggests that the HEPC acts on the release of pro‐inflammatory kinins, reducing the oedema. Also, we could observe that the PC meloxicam, in 1 and 3 mg/kg dosages, presented significant statistics (*p* < 0.005) in only 60 min.

Dextran sulphate can activate the Hageman factor (factor XII of coagulation), which is responsible for the formation of kininogen [36]. In this context, factor XII and prekallikrein can be activated in the presence of kininogen, resulting in the cleavage of kininogen by kallikrein and the release of bradykinin, a neurotransmitter that induces vasodilation and increased vascular permeability [35]. In our findings, the increase in permeability caused by DEX was significantly inhibited by HEPC, suggesting the involvement of kinins in this inhibition. HEPC may act either on kinin or on oedema through bradykinin receptors.

#### 2.2.4 Paw Oedema Induced by Compound 4880

**FIGURE 5:**
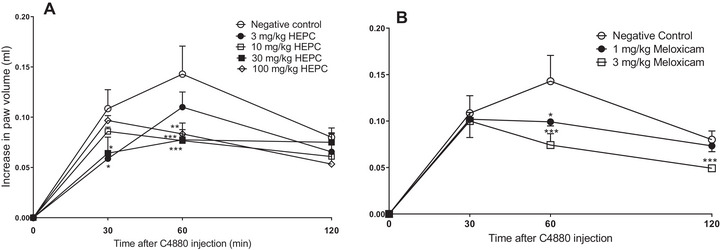
Mean inhibitory effect of the hydroalcoholic extract of *Persea cordata* (HEPC, A) and meloxicam (B) on paw oedema induced by compound 4880 in the hind limb of the mice. Each group represents the mean of five to six animals, and the vertical lines indicate the SEM. Significantly different from the negative control (NC), *p < 0.05, **p < 0.001, and ***p < 0.0001, in Dunnett's test.

In the paw oedema induced by C4880, it was observed that HEPC showed significant statistical inhibition only in 60 min (Figure 5A), (*p* < 0.0001) at doses of 10 and 30 mg/kg, and statistical significance (*p* < 0.001) at the dose of 100 mg/kg at the same time.

HEPC at doses of 3, 30, and 100 mg/kg showed the highest effect at 30, 60, and 120 min with MI values of 52.27 ± 6.28%, 61.03 ± 3.61%, and 72.76 ± 6.08%, respectively.

Meloxicam at a dose of 3 mg/kg inhibited oedema at all times with MI values of 53.27 ± 6.28%, 62.53 ± 7.58%, and 73.73 ± 1.81%, respectively. It can be observed that HEPC and PC have similar inhibition percentages.

C4880 is a classic agent for the study of anti‐inflammatory drugs, acting as a serotonin and histamine releaser from mast cells, which are responsible for oedema formation [37, 38]. The oedema induced by this phlogistic agent was inhibited by HEPC, suggesting the involvement of this phenomenon in our study. Histamine increases microvascular leakage via H1 and H2 receptors [39]. This reduction in paw oedema induced by C4880 suggests that the prevention of cellular degranulation from mast cells may contribute to its anti‐oedematogenic effect in the early stages of the inflammatory process.

#### 2.2.5 Nociception Induced by Intraperitoneal Injection of Acetic Acid in Mice

**FIGURE 6:**
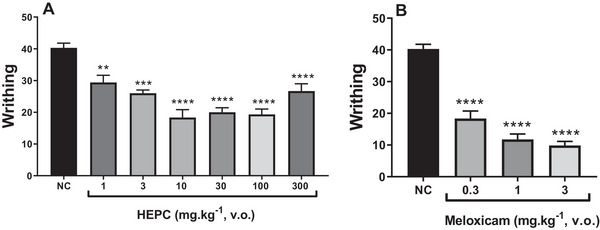
Antinociceptive effect of the hydroalcoholic extract of *Persea cordata* (HEPC, A) and meloxicam (B) on acetic acid‐induced abdominal writhing in mice. Each group represents the mean of five to six animals, and the vertical lines indicate the SEM. Significantly different from the negative control (NC): **p < 0.01; ***p < 0.001; ****p < 0.0001 in Dunnett's multiple comparison test.

The results presented in Figure 6 indicate that orally administered HEPC (1–100 mg/kg) showed a significant dose‐dependent inhibition of acetic acid‐induced abdominal writhing compared to the NC (Figure 6A). The 10 mg/kg dose exhibited the highest statistical significance (*p* < 0.0001) when compared to meloxicam (Figure 6B).

Paradoxically, at the 300 mg/kg dose of HEPC, an agonistic effect was observed with a reduction in analgesic activity, counteracting the analgesic effect previously observed at doses of 10–100 mg/kg. This may be due to the specific characteristics of the injured tissue and the inflammatory/algogenic soup mediators involved.

In the study of nociception, abdominal writhing, formalin and hot plate tests were used to investigate the potential analgesic mechanisms of action. In the writhing test, HEPC exhibited a significant and dose‐dependent inhibition of abdominal constrictions, with approximately 63% inhibition observed at the oral dose of 10 mg/kg. However, at the dose of 300 mg/kg, HEPC exhibited a paradoxical agonistic effect, with a reduction in its analgesic activity. In general, acetic acid induces pain through the release of endogenous mediators such as serotonin, histamine, prostaglandins and pro‐inflammatory cytokines like IL‐1β and TNFα, by modulating macrophages and mast cells located in the peritoneal cavity [40]. The results observed in this model demonstrated a potent antinociceptive effect, and the likely mechanism underlying this effect is the reduction in the synthesis or release of inflammatory mediators. Although the writhing test lacks specificity, it is a highly sensitive method for screening compounds with antinociceptive properties [41].

#### 2.2.6 Nociception Induced by Intraplantar Formalin Injection in Mice

To confirm the analgesic effect of HEPC, the formalin test was employed. This pain model exhibits a biphasic response: an immediate phase (0–5 min), characterized by intense neurogenic pain resulting from the direct chemical stimulation of nociceptive afferent fibres, primarily C fibres, and the release of substance P [42]. This is followed by the late phase (20–25 min), which corresponds to inflammatory pain caused by the release of serotonin, histamine, bradykinin, and prostaglandins [43]. Drugs that act primarily as central analgesics usually inhibit both phases, whereas peripherally acting drugs inhibit only the second phase [44].

**FIGURE 7:**
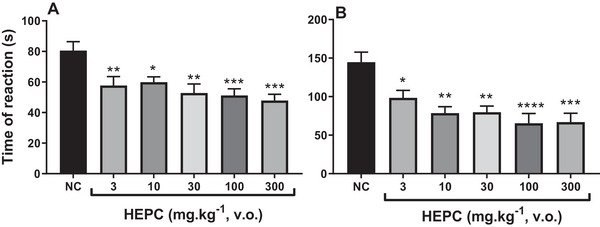
The effect of the hydroalcoholic extract of *Persea cordata* (HEPC) administered orally on the first (A) and second (B) phase of formalin‐induced pain. Each group represents the mean of five to six animals, and the vertical lines indicate the SEM (standard error of the mean). Significantly different from the negative control (NC): *p < 0.05; **p < 0.01; ***p < 0.001; ****p < 0.0001 in Dunnett's multiple comparison test.

The results presented in Figure 7 show that HEPC (3–300 mg/kg) caused significant inhibition of both phases of formalin‐induced pain, although it was more effective during the second phase of this model.

In the first phase, the maximum inhibition by HEPC averaged 59.45 ± 4.89% (300 mg/kg, po), with marked statistical significance compared to the NC (*p* < 0.001) (Figure 7). This initial phase appears to be predominantly caused by the direct chemical stimulation of nociceptors, specifically the activation of C fibres and, to a lesser extent, Aδ fibres. Therefore, this phase is mainly associated with neurogenic mechanisms. Algogenic mediators such as glutamate, substance P, and bradykinin are released [45, 46]. At the same time, other sensory neuropeptides released via axon reflexes activate neutrophils, fibroblasts and mast cells [47].

**FIGURE 8:**
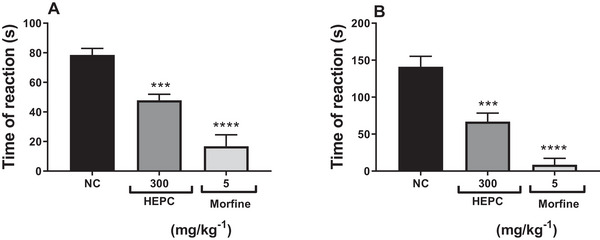
Effect of the hydroalcoholic extract of *Persea cordata* (HEPC, oral route) and morphine (subcutaneous route) on the first (A) and second (B) phase of formalin‐induced pain. Each group represents the mean of five to six animals, and the vertical lines indicate the SEM. Significantly different from the negative control (NC): ***p < 0.001; ****p < 0.0001 in Dunnett's test.

In the second phase, which is considered inflammatory, HEPC showed a maximum reduction in pain behaviour (licking time) of 67.01 ± 5.23% at the dose of 100 mg/kg (*p* < 0.0001). The late phase is believed to depend on a combination of an inflammatory reaction in peripheral tissue, involving prostaglandins, serotonin, histamine and bradykinin, among other mediators [45, 48, 49]. Thus, it may be inferred that the antinociceptive effect of HEPC is likely mediated by a central mechanism, as it inhibited both phases of the formalin test, although with lower efficacy and potency than morphine, as shown in Figure 8.

In this experimental model, morphine was used as a positive control (PC) drug in the formalin test, as it inhibits both phases of the test [48, 49]. Therefore, the administration of morphine (5 mg/kg, sc) in the neurogenic phase (Figure 8A) reduced pain behaviour by 82.97 ± 6.99%, and in the inflammatory phase (Figure 8B), the inhibition rate was 95.25 ± 4.75%.

#### 2.2.7 Hot Plate Test

**FIGURE 9:**
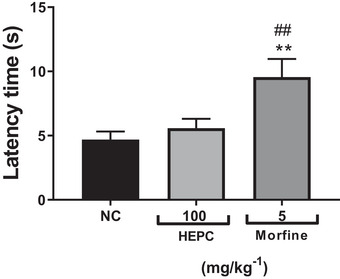
Effect of the ethanolic extract of *Persea cordata* and morphine in the hot plate test in mice. Data are expressed as mean ± SEM for latency time in seconds on the hot plate (*n* = 6–8 animals per group). Statistically significant according to Dunnett's multiple comparison test (**p < 0.01) and Newman–Keuls test (^##^ *p* < 0.01) versus negative control group (NC). The hydroalcoholic extract of *P. cordata* (HEPC) was administered orally, and morphine was administered subcutaneously.

Thus, to confirm the antinociceptive effect, the hot plate test was used, which helps support a possible involvement of HEPC in central analgesic activity. This test is commonly used in analgesia studies to assess the response to a thermal stimulus mediated by central integration [50]. It reflects the activity of temperature‐sensitive Aδ and C afferent fibres, with substance P participating as a transmitter in the afferent pathways of the spinal cord. It is used to detect both narcotic and non‐narcotic analgesics, as well as to distinguish analgesic properties from anti‐inflammatory properties. It is well established that thermal nociceptive tests are more sensitive to μ‐opioid agonists, while non‐thermal tests are more sensitive to κ‐opioid agonists [51, 52]. HEPC did not significantly increase the latency time in response to the thermal stimulus in the test, ruling out a possible central influence on its antinociceptive effect. The hot plate test is a classic nociception model in rodents, and the response pattern observed in this study showed that the mean latency time of the NC group was 4.69 ± 0.63 s (Figure 9). However, the group treated with HEPC at the dose of 100 mg/kg did not show a significant increase in latency time compared to the control group when evaluated in the thermal nociception model using the hot plate test, under conditions in which morphine produced a significant antinociceptive effect (*p* < 0.005), with a mean latency time of 9.55 ± 1.41 s (Figure 9).

#### 2.2.8 Motor Performance in the Rota‐Rod Apparatus

**FIGURE 10:**
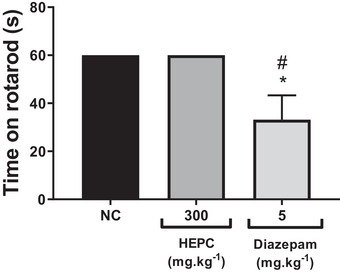
Motor performance in the rota‐rod apparatus in mice treated with hydroalcoholic extract of *Persea cordata* (HEPC, oral route) and diazepam (intraperitoneal route) versus the negative control group. Data are expressed as mean ± S.E.M. for walking duration on the rota‐rod apparatus (*n* = 5–6 animals per group). Statistically significant according to Dunnett's multiple comparison test (*p < 0.05) and Newman–Keuls test (^#^ *p* < 0.05) versus negative control group (NC).

The rotarod test is performed to assess the motor performance of mice and to investigate whether there is sedation resulting from new drugs or compounds in preclinical trials [53]. As expected, the control group treated with phosphate‐buffered saline (PBS) (po) remained walking on the apparatus for 60 s without falling from the rotating bar. HEPC at 300 mg/kg (po) did not alter motor performance, as the time spent on the rota‐rod by the treated group was the same as that of the control group (Figure 10). Furthermore, the animals did not show signs of prostration or increased sleep time in either the control or extract‐treated group. Therefore, the antinociceptive effect of HEPC observed in the pain models used is not associated with motor impairment and/or depression of motor coordination, suggesting it is not related to central depressant effects.

Therapeutic properties of *P. cordata* as a medicinal plant acting through different mechanisms of action to produce anti‐inflammatory effects may rely on the predominant compounds, including flavonoids (sakuraresinol, procyanidin B2 rutinoside, procyanidin B5 rutinoside and naringin dihydrochalcone), lignan (lyoniside and ssioriside) and diterpene glucosides (sagittarioside b). In a previous study, naringin dihydrochalcone showed significantly improved lung damage by modulation pro‐inflammatory cytokine levels (TNF‐α, IL‐6, IL‐1β) in both cells and lung tissues. This property occurs by attenuating the activation of STAT3, NF‐κB and NLRP3 inflammatory signalling pathways [54]. Lyoniside displayed neuroprotective effects against glutamate‐induced neurotoxicity in HT22 cells [55]. Although no reports were found on analgesic and anti‐inflammatory effects of procyanidin B2 rutinoside, the aglycone (procyanidin B2) showed in previous studies anti‐inflammatory through inhibition of IL‐1β and IL‐18 [56]. Lyoniside reduced in a dose dependent manner the level of nitric oxide and IL‐6 in LPS‐induced RAW264.7 cells [57]. The hydroalcoholic crude extract displayed analgesic effect at 10 and 100 mg/kg and in contrast this property was less expressive at the highest dose. Studies revealed that phthalates exposure causes inflammation through the increase of mediators such as IL‐1β, IL‐10, IL‐17A, interferon‐γ and TNF‐α. [58, 59] At a dose of 300 mg/kg, the phthalate concentration must be at the amount necessary to trigger inflammation and counteract the expected effect, while at low doses its effect would be limited by its low concentration.

## 3 Conclusions

The results suggest that HEPC exhibits a non‐specific anti‐oedematogenic effect via oral route administration when challenged with different phlogistic agents that induce oedema. This inhibitory effect on the oedema was long‐lasting, persisting for up to 24 h after challenge with CAR and PCS.

HEPC demonstrated a non‐specific peripheral antinociceptive effect by inhibiting pain‐related behaviour in the abdominal writhing and formalin tests. Therefore, these findings indicate the potential of this plant extract for the treatment of painful and inflammatory conditions. The antinociceptive effect of HEPC does not appear to be related to central mechanisms of action, as no sedation or muscle relaxation was observed in the motor performance test. Similarly, in the hot plate assay, the extract did not increase the latency time in response to the thermal stimulus, suggesting that the analgesic effect is not mediated by the opioid system.

Thus, these results support previous findings regarding the plant's anti‐inflammatory and analgesic potential and validate the ethnomedical use of the tree bark as a popular remedy.

## 4 Experimental Section

### 4.1 Materials

UPLCMS analysis was performed on a quadrupole orthogonal acceleration time‐of‐flight (Q‐TOF) mass spectrometer Xevo GS‐2 QTof equipped with an electrospray ionization (ESI) source. The mass device is coupled with an Acquity H‐Class UPLC system (Waters Co., USA) composed of an auto sampler, quaternary solvent manager, and column oven. All solvents (LCMS grade) were purchased from Servylab, a local provider of Sigma‐Aldrich (São Leopoldo, Rio Grande do Sul, Brazil). Solvents used for extraction were purchased from Rauter (Gravataí, Rio Grande do Sul, Brazil)

### 4.2 Ethics in Animal Use

The study was submitted to and approved by the Animal Ethics Committee (CEUA) of the Federal University of Fronteira Sul (UFFS), under protocol numbers CEUA No. 4477180519 and No. 4288121019, in accordance with the ethical principles of animal welfare established by the National Council for the Control of Animal Experimentation (CONCEA).

### 4.3 Plant Material and Preparation of the Hydroalcoholic Extract

The botanical identification voucher specimen of *P. cordata* was deposited in the herbarium of the Centre for Agricultural and Veterinary Sciences (CAV) at the State University of Santa Catarina (UDESC), in Lages, SC, under No. 045, in 2002, by Claudete Scharge Nuernberg, PhD.

The bark of *P. cordata* was collected in Bom Retiro, Santa Catarina, in September 2021. It was obtained in its natural state from well‐preserved remnants of the Araucaria Forest, far from human contact. The bark was carefully harvested, taking special care to avoid girdling and to prevent damage to the tree.

The bark of *P. cordata* was ground and macerated for 7 days in ethanol:water (7:3) in a proportion of 100 g of plant material in 1 L of solvent. The crude extract was obtained by filtration and rotary evaporation in vacuo. The dried extract (HEPC) was stored in tightly sealed containers and kept frozen at −20°C for further use.

An aliquot of HEPC was dissolved in 5% dimethyl sulfoxide (DMSO) and subsequently diluted in PBS to reach the desired concentration used for administration to the animals.

### 4.4 Ultra‐Performance Liquid Chromatography Coupled to Electrospray Ionization High Resolution Mass Spectrometry (UPLC–ESI–HRMS) Method

The chromatographic separation was performed using a chromatographic column Acquity UPLC BEH C18 (50 × 2.1 mm i.d., 1.7 µm) at 40°C. A gradient system composed of 0.1% aqueous formic acid (pH 3.0) (A), ANC (B) at a flow rate of 0.3 mL/min. Elution conditions were as follow: 0 min 90% (A), 10% (B), 0‐1 min 80% (A), 20% (B); 1–12 min 10% (A), 90% (B), 12–15 min 10% (A), 90% (B). The proportion was maintained for 5 min. The sample was injected at a volume of 0.3 µL.

MS and MS^e^ data (in two scan functions) were acquired in centroid mode with a scan time of 1 s, and a mass range of 50–1500 Da. Collision energy was set from 20 to 35 eV. Accurate mass values were obtained by correction during acquisition with the external reference (LockSpray) leucine enkephalin solution (1 ng/mL, at 20 µL/min).

Positive mode of ionisation used a capillary voltage of 2.5 kV, sampling cone voltage of 40 V, source offset voltage of 80 V, desolvation temperature of 250°C, source temperature of 90°C, cone gas flow of 180 L/h and desolvation gas flow of 600 L/h.

Negative mode of ionisation used a capillary voltage of 2.5 kV, sampling cone voltage of 40 V, source offset voltage of 80 V, desolvation temperature of 350°C, source temperature of 150°C, cone gas flow of 100 L/h and desolvation gas flow of 250 L/h. Data processing was performed using Mass Lynx 4.1 software (Waters Co.).

### 4.5 Animals

Albino Swiss mice (Mus musculus), with an average age of 3 months (25–30 g), were used in the experiments. The animals were obtained from the Central Animal Facility of UFFS—Realeza Campus. They were housed in a temperature‐controlled environment (23 ± 2°C) under a 12‐h light/dark cycle, with free access to food and water (ad libitum). The animals were transferred to the laboratory 12 h before the experiments to allow for acclimatization to the new environment.

### 4.6 Biological Assays

#### 4.6.1 Paw Oedema Test

The anti‐oedematogenic effect of HEPC was carried out using the ‘mouse paw oedema’ model described by Winter et al. [60].

Animals from different groups (*N* = 5–6), randomly selected, were treated 1 h before the experiments with four increasing doses of HEPC (3, 10, 30, and 100 mg/kg, po). PC groups received meloxicam at doses of 1 and 3 mg/kg (po), and the NC group received only PBS (0.1 mL/10 g, po).

After treatment, intraplantar administration of phlogistic agents was performed subcutaneously into the plantar cushion of the right hind paw using a 0.5 mL syringe with a 30G (8 × 0.3 mm) needle. As a contralateral control, PBS was injected into the opposite paw. The animals were challenged with the following phlogistic agents: CAR (300 µg/paw), capsaicin (PCS, 3 µg/paw), dextran (DEX, 100 µg/paw), and compound 4880 (C4880, 12 µg/paw), diluted in 50 µL of PBS and administered promptly during mechanical restraint.

The measurement of oedema was performed using a plethysmometer (EFF 304, Insight), in which the volume of liquid displaced by immersing the animals' paws in a polypropylene cuvette was observed and recorded on a digital display. The limb was immersed up to the lateral malleolus of the heel in the cuvette containing 5% sodium lauryl ether sulphate solution. Thus, the amount of liquid displaced in the cuvette, measured in microliters (µL), was considered the volume corresponding to paw oedema. The final oedema volume was expressed as the difference between the right and left hind paws.

Oedema induced by the phlogistic agents CAR and PCS was evaluated at six different moments (M): M1—30 min after application; M2—60 min after application; M3—120 min after application; M4—240 min after application; M5—12 h; and finally, M6—24 h after the administration of the phlogistic agents into the animal's paw. For the measurement of oedema induced by DEX, readings were taken up to M4, and for C4880, up to T3.

### 4.7 Antinociceptive Activity

#### 4.7.1 Nociception Induced by Intraperitoneal Injection of Acetic Acid in Mice

This assay aimed to evaluate visceral pain. The induction of abdominal writhing was performed by intraperitoneal administration of 0.6% acetic acid solution, at a dose of 0.16 mL/10 g of mouse body weight, 1 h after treatment of the respective groups: NC (PBS: 0.1 mL/10 g, po), PC (meloxicam, 0.3, 1, and 3 mg/kg, po) and HEPC (1, 3, 10, 30, 100, or 300 mg/kg, po). The animals were placed in individual glass funnels during the observation period. Writhing was indicated by abdominal constriction and full extension of the hind limbs. Ten minutes after acetic acid administration, the number of abdominal writhes was counted over 20 min [61].

#### 4.7.2 Nociception Induced by Intraplantar Injection of Formalin in Mice

The formalin test was used to assess acute and tonic pain, according to the protocol described by Hunskaar and Hole [45]. This test presents two distinct phases: the acute or neurogenic phase (0–5 min) and the late or inflammatory phase (15–30 min) [62]. The formalin assay can be considered a short‐term inflammatory pain model, involving moderate and continuous pain resulting from tissue injury [63]. A 2.5% formaldehyde solution was prepared in PBS to obtain the formalin solution. The animals were treated with vehicle (PBS: 0.1 mL/10 g, po), HEPC (10, 30, 100, or 300 mg/kg, po), and the PC group received morphine (5 mg/kg, sc), 1 h before the subcutaneous injection of 20 µL of 2.5% formalin into the plantar surface of the left hind paw. The mice were placed in individual glass funnels for observation. Pain responses were evaluated based on behaviours such as licking or biting of the injected paw, which were timed in seconds using a digital stopwatch. The measurement was divided into two time periods: the early phase, which lasts the first 5 min, and the late phase, which occurs from 15 to 30 min after formalin injection [45, 62].

#### 4.7.3 Hot Plate Test

The hot plate test was used to measure response latency to a thermonociceptive stimulus, according to the method described by Eddy and Leimbach [64]. This test is specific for compounds that possess opioid‐type analgesic activity. The time the animals remained on a heated metal plate (55 ± 0.5°C) was evaluated until they showed a reaction to the thermal stimulus (interpreted as jumping, raising, or licking the paws or hands) [65]. A cut‐off time of 20 s was established, meaning the maximum allowed time on the heated plate was 20 s, to avoid tissue damage. Treated animals received HEPC (100 mg/kg, po), while the NC group received the vehicle PBS (0.1 mL/10 g, po). Morphine, administered subcutaneously at a dose of 5 mg/kg, was used as the PC. Recordings were taken 60 min after administration of HEPC, vehicle, and reference drug. Latency time in seconds was recorded using a digital stopwatch. Animals were immediately removed as soon as a response was observed.

The formula used to determine the percentage of time the animals remained on the hot plate was percentage of maximum effect (PME) = (FT − IT)/(20 − IT) × 100 adapted from the method reported by Aanonsen and Wilcox [66] where FT is the final time and IT is the initial time.

#### 4.7.4 Effect on Motor Performance in the Rota‐rod Model

The rota‐rod apparatus consists of a horizontal rotating rod, suspended at a height of 25–30 cm and rotating at a constant speed (20 rpm). This is a sensorimotor performance test, carried out to evaluate potential muscle relaxant and central sedative effects of drugs [67]. The animals were trained 24 h before the experiments for a period of 3–5 min. Mice that remained on the rotating bar of the rota‐rod apparatus for at least two consecutive 60‐s intervals were selected. Motor coordination was assessed based on the time the mouse remained walking on the rotating rod for a maximum period of 60 s. The following treatments were administered: HEPC (300 mg/kg, po), vehicle (PBS 0.1 mL/10 g, po) in the NC group, and diazepam (5 mg/kg, ip) in the PC group. Sixty minutes after drug administration, the test was initiated.

#### 4.7.5 Drugs and Reagents

The drugs used for biological assays were capsaicin, lambda CAR, compound 4880, DMSO, dextran sulphate, diazepam, meloxicam, morphine, and naloxone (all from Sigma‐Aldrich), and PBS (containing 137 mM NaCl, 2.7 mM KCl, and 10 mM phosphate buffer), which was used as the vehicle for drug dilution. All other solutions used were of analytical grade.

#### 4.7.6 Statistical Analysis

The results were expressed as mean ± standard error of the mean (SEM) and analysed using GraphPad Prism version 8.0. Data were subjected to two‐way analysis of variance (ANOVA), followed by Dunnett's multiple comparison test. Probability differences of less than 5% (*p* < 0.05) were considered statistically significant.

## Author Contributions

**Valfredo Schlemper**: conceptualization, methodology, investigation, writing – original draft preparation, supervision, funding acquisition. **Daniela Hemsing**: conceptualization, investigation. **Julia Elisabett K. Bolsonello**: methodology, investigation. **Susana R. de Mello Schlemper**: methodology. **Tiago Tizziani**: methodology. **Lutuima A. Capangue Neto**: methodology. **Louis P. Sandjo**: conceptualization, methodology, investigation, writing – original draft preparation, supervision, funding acquisition. All authors have read and agreed to the published version of the manuscript.

## Conflicts of Interest

The authors declare no conflicts of interest.

## Data Availability

The data that support the findings of this study are available from the corresponding author upon reasonable request.
